# Metabolomic insights into variable antihistamine responses in allergic rhinitis: unveiling biomarkers for precision treatment

**DOI:** 10.3389/fimmu.2025.1565972

**Published:** 2025-06-17

**Authors:** Xiaohong Lyu, Yi Liu, Hongna Li, Zhoujie Wu, Yi Sun, Xuehan Jiang, Shandong Wu, Shanhong Wu, Rui Tang, Yue Gao, Jinlyu Sun

**Affiliations:** ^1^ Department of Allergy, Beijing Key Laboratory of Precision Medicine for Diagnosis and Treatment of Allergic Diseases, National Clinical Research Center for Dermatologic and Immunologic Diseases, Peking Union Medical College Hospital, Chinese Academy of Medical Sciences, Peking Union Medical College, Beijing, China; ^2^ Department of Breast Surgery, Peking Union Medical College Hospital, Chinese Academy of Medical Sciences, Peking Union Medical College, Beijing, China; ^3^ Hangzhou Zheda Dixun Biological Gene Engineering Co.,Ltd., Hangzhou, China; ^4^ Pulmonary and Critical Care Medicine, Binzhou People’s Hospital, Binzhou, China; ^5^ Zhejiang Key Laboratory of Traditional Chinese Medicine for the Prevention and Treatment of Senile Chronic Diseases, Affiliated Hangzhou First People’s Hospital, School of Medicine, Westlake University, Hangzhou, China

**Keywords:** allergic rhinitis, metabolomics, treatment response, biomarkers, LC-MS

## Abstract

**Background:**

The clinical response to antihistamine therapy exhibits substantial heterogeneity among individuals with allergic rhinitis (AR). While these medications represent a cornerstone in AR management, the molecular basis underlying differential treatment outcomes remains incompletely understood. This investigation sought to delineate specific metabolomic profiles that distinguish between AR patients who demonstrate favorable responses to antihistamine treatment and those who exhibit therapeutic resistance.

**Methods:**

This investigation encompassed a cohort of 57 patients diagnosed with AR, stratified into antihistamine-effective (n=49) and antihistamine-ineffective (n=8) groups. The study protocol integrated multiple analytical approaches, including clinical phenotyping, serum vitamin D quantification, mRNA expression, and untargeted metabolomic analysis. Metabolomic profiling was conducted using a state-of-the-art liquid chromatography-mass spectrometry (LC-MS) platform, enabling comprehensive characterization of the serum metabolome.

**Results:**

While demographic characteristics and vitamin D levels showed no significant differences between two groups, blood H1R mRNA expression was significantly higher in antihistamine-ineffective patients (P=0.046), and nasal TPSB mRNA expression was elevated (P=0.006). Nineteen metabolites showed significant differences (p<0.05, fold change>2.0, VIP>1.0) between groups. ROC curve analysis identified nine metabolites with high diagnostic potential (AUC>0.70), with Methotrexate (AUC=0.862), Pro-Val-Ala-Glu-Val (AUC=0.804), and TyrMe-Ile-OH (AUC=0.791) showing the strongest discriminatory power. Pathway analysis highlighted the involvement of caffeine metabolism and tryptophan metabolism pathways.

**Conclusions:**

This study identified distinct metabolomic signatures between antihistamine-effective and antihistamine-ineffective AR patients, providing potential biomarkers for predicting treatment response and new insights into the metabolic mechanisms underlying treatment efficacy in AR.

## Introduction

Allergic rhinitis (AR) is a prevalent chronic inflammatory condition affecting the nasal mucosa, with an estimated global prevalence of 10-30% (1). This disorder not only disrupts patients’ quality of life but also imposes a considerable socioeconomic burden (2). AR is an IgE-mediated inflammation of the nasal mucosa, presenting with symptoms such as rhinorrhea, nasal congestion, sneezing, and itching. Although a range of treatments, including antihistamines and allergen immunotherapy, are available, patient responses to these therapies vary widely. While some individuals achieve significant symptom relief, others experience limited improvement (3). This variability underscores the pressing need for predictive biomarkers to enable more personalized and effective treatment strategies.

Understanding the mechanisms underlying variable treatment responses is essential for advancing personalized therapeutic strategies. Metabolomics, an emerging discipline within systems biology, provides a powerful platform to explore metabolic changes linked to disease states and therapeutic outcomes (4). While this approach has demonstrated potential in several allergic disorders, its application in deciphering treatment efficacy in AR remains relatively underexplored (5). Recent advancements in high-throughput metabolomics, particularly liquid chromatography-mass spectrometry (LC-MS), have made it possible to analyze thousands of metabolites simultaneously, offering valuable insights into disease pathways (6) and treatment responses (7). Despite these technological strides, limited research has focused on comparing metabolomic profiles between AR patients who respond effectively to treatment and those who do not, leaving a critical gap in understanding the variability in treatment outcomes.

Precision medicine in allergic diseases has underscored the need for reliable biomarkers to enable patient stratification and optimize treatment selection (8). Although traditional clinical parameters and biomarkers, such as total IgE levels, have been employed, their predictive value for treatment outcomes remains limited. Combining metabolomic profiling with clinical and molecular data offers a novel and promising strategy to gain deeper insights into the mechanisms driving variability in treatment responses.

This study aimed to identify metabolomic signatures capable of distinguishing between antihistamine-effective and antihistamine-ineffective AR patients. By integrating comprehensive metabolomic profiling with clinical characteristics, vitamin D levels, and RNA expression analysis, we sought to establish a more reliable method for predicting treatment outcomes in AR patients. This research marks a significant step toward advancing personalized medicine in AR management, paving the way for more targeted and effective therapeutic strategies.

## Methods

### Study population and design

This study was carried out at Peking Union Medical College Hospital in Beijing between October 2023 and January 2024. The study protocol received approval from the institutional ethics committee of Peking Union Medical College Hospital (approval number: K3949), and written informed consent was obtained from all participants.

### Diagnosis and treatment response definition

AR was diagnosed following the Allergic Rhinitis and its Impact on Asthma (ARIA) guidelines (2, 9), based on typical symptoms, positive specific IgE tests, and skin prick test results. To specifically assess the effectiveness of antihistamine treatment while minimizing potential confounding factors, all enrolled patients, after providing informed consent, were treated with antihistamines for 4 weeks without the use of intranasal corticosteroids.

Treatment effectiveness was defined as follows: patients were classified as antihistamine-effective if they achieved a ≥50% reduction in the total nasal symptom score (TNSS) (TNSS) (10, 11) after 4 weeks of treatment, and as antihistamine-ineffective if the reduction in TNSS was <50%. TNSS was calculated by summing the scores of four individual symptoms—rhinorrhea, nasal congestion, nasal itching, and sneezing—each rated on a scale from 0 to 3 (0 = none, 1 = mild, 2 = moderate, 3 = severe).

The exclusion criteria for all groups included a history of autoimmune diseases, the use of immunosuppressive medications, or antibiotic use within the previous 4 weeks. Additionally, patients with a history of allergen-specific immunotherapy within the past 5 years were excluded to avoid potential confounding effects on the metabolomic profile (2). Those requiring systemic corticosteroids for AR management were also excluded from the study.

### Clinical assessment

Demographic data and clinical characteristics were gathered using standardized questionnaires. Seasonal allergies and sensitivities to specific allergens were assessed through clinical history, standard skin prick tests, and serum-specific IgE measurements. Additionally, a family history of allergies was recorded for all participants.

### Vitamin D analysis

Serum vitamin D levels, including total vitamin D, vitamin D2, and vitamin D3, were measured using liquid chromatography-tandem mass spectrometry (LC-MS/MS) in accordance with standardized protocols.

### mRNA expression analysis

mRNA was extracted from blood samples and nasal swabs using the Fluorescent PCR-chip method (Hangzhou Zheda Dixun Biological Gene Engineering Co., Ltd.). The analysis included the expression levels of eosinophil cationic protein (ECP), beta-tryptase-like protein (TPSB), cysteinyl leukotriene type 1 receptor (CYS1), CYS2, histamine type 1 receptor (H1R), and histamine type 4 receptor (H4R). Results were normalized to housekeeping genes and expressed as copies/µL.

### Metabolomic sample preparation and extraction

#### Liquid samples class I

Blood samples were collected in serum separator tubes following the recommended handling guidelines for metabolomics studies. Serum was separated and stored at -80°C within 2 hours of collection. Prior to analysis, the samples stored at -80°C were thawed on ice and vortexed for 10 seconds. A 50 μL aliquot of the sample was mixed with 300 μL of extraction solution (ACN: Methanol = 1:4, v/v) containing internal standards in a 2 mL microcentrifuge tube. The mixture was vortexed for 3 minutes and centrifuged at 12,000 rpm for 10 minutes at 4°C. Subsequently, 200 μL of the supernatant was collected, placed at -20°C for 30 minutes, and centrifuged again at 12,000 rpm for 3 minutes at 4°C. Finally, 180 μL of the supernatant was transferred for LC-MS analysis.

#### Metabolomic profiling

Non-targeted global metabolomic profiles were generated using ultra-performance liquid chromatography (LC-30A, Shimadzu, Japan) coupled with a high-resolution/accurate mass spectrometer (TripleTOF 6600+, SCIEX, Foster City, CA, USA) in an LC-MS/MS setup. All samples were analyzed using two LC/MS methods. One aliquot was analyzed under positive ion conditions and eluted from a T3 column (Waters ACQUITY Premier HSS T3 Column, 1.8 µm, 2.1 mm × 100 mm) with 0.1% formic acid in water as solvent A and 0.1% formic acid in acetonitrile as solvent B. The gradient used was as follows: 5% to 20% solvent B in 2 minutes, increased to 60% in the next 3 minutes, further increased to 99% in 1 minute and held for 1.5 minutes, then returned to 5% solvent B within 0.1 minute and held for 2.4 minutes. The analytical conditions were as follows: column temperature, 40°C; flow rate, 0.4 mL/min; injection volume, 4 μL. A second aliquot was analyzed under negative ion conditions, following the same elution gradient as in the positive ion mode.

### MS conditions (AB)

Data acquisition was performed using the information-dependent acquisition (IDA) mode with Analyst TF 1.7.1 software (Sciex, Concord, ON, Canada). The source parameters were set as follows: ion source gas 1 (GAS1), 50 psi; ion source gas 2 (GAS2), 50 psi; curtain gas (CUR), 25 psi; temperature (TEM), 550°C; declustering potential (DP), 60 V for positive mode and −60 V for negative mode; and ion spray voltage floating (ISVF), 5000 V for positive mode and −4000 V for negative mode.

The TOF MS scan parameters were configured as follows: mass range, 50–1000 Da; accumulation time, 200 ms; and dynamic background subtraction, enabled. The product ion scan parameters were set as follows: mass range, 25–1000 Da; accumulation time, 40 ms; collision energy, 30 V for positive mode and −30 V for negative mode; collision energy spread, 15; resolution, UNIT; charge state, 1 to 1; intensity threshold, 100 cps; exclusion of isotopes within a 4 Da window; mass tolerance, 50 ppm; and a maximum of 18 candidate ions to monitor per cycle.

### Statistical analysis

The raw data files acquired by LC-MS were converted into mzXML format using ProteoWizard software. Peak extraction, alignment, and retention time correction were conducted using the XCMS program. The “SVR” method was applied to correct peak areas, and peaks with a detection rate lower than 50% in each sample group were excluded. Metabolite identification was then performed by referencing the laboratory’s self-built database, integrated public databases, AI databases, and metDNA.

Unsupervised principal component analysis (PCA) was conducted using the prcomp function in R (www.r-project.org), with the data scaled to unit variance prior to analysis. Hierarchical cluster analysis (HCA) results for samples and metabolites were visualized as heatmaps with dendrograms, while Pearson correlation coefficients (PCC) between samples were calculated using the cor function in R and displayed as heatmaps. Both HCA and PCC analyses were performed using the R package ComplexHeatmap. For HCA, normalized signal intensities of metabolites (unit variance scaling) were represented as a color spectrum in the heatmaps.

For two-group analysis, differential metabolites were identified based on the following criteria: VIP > 1, P-value < 0.05 (Student’s t-test), and fold change > 2.0. VIP values were extracted from the OPLS-DA results, which included score plots and permutation plots, and were generated using the R package MetaboAnalystR. Prior to OPLS-DA analysis, the data were log-transformed (log2) and mean-centered. To prevent overfitting, a permutation test with 200 iterations was conducted.

Identified metabolites were annotated using the KEGG Compound database (http://www.kegg.jp/kegg/compound/), and the annotated metabolites were subsequently mapped to the KEGG Pathway database (http://www.kegg.jp/kegg/pathway.html). Significantly enriched pathways were identified using a hypergeometric test based on the P-value for the given list of metabolites.

The following R packages were utilized in this study: base (version 4.1.2), corrplot (version 0.92), ComplexHeatmap (version 2.9.4), MetaboAnalystR (version 1.0.1), fmsb (version 0.7.1), igraph (version 1.2.11), ggraph (version 2.0.5), and FELLA (version 1.2.0).

## Results

A total of 57 patients with allergic rhinitis were included in the study, consisting of 49 cases responsive to antihistamines and 8 cases unresponsive to antihistamines (**Table 1**). The mean age of patients in the antihistamine-effective group was 33.88 ± 8.40 years, compared to 29.25 ± 9.56 years in the antihistamine-ineffective group (P = 0.162). The proportion of male patients was similar between the two groups (28.6% *vs*. 25.0%, P = 0.835). The mean rhinitis scores were also comparable (2.79 ± 0.50 *vs*. 2.88 ± 0.35, P = 0.627).

**Table 1:** Summary characteristics of the study population.

Characteristics	Antihistamine-effective group (n=49)	Antihistamine-ineffective group (n=8)	P-value*
Age, year	33.88 ± 8.40	29.25 ± 9.56	0.162
Sex, male, n, %	14 (28.6%)	2 (25.0%)	0.835
Rhinitis Score	2.79 ± 0.50	2.88 ± 0.35	0.627
Maximum medical history, years	7.87 ± 6.51	5.12 ± 3.87	0.254
Seasonal allergies	38 (79.2%)	7 (87.5%)	0.583
Allergy to specific allergens	16 (32.7%)	0 (0.0%)	0.057
Family history of allergies	19 (38.8%)	2 (25.0%)	0.454

* suggests p<0.05 for statistically different results.

The maximum duration of medical history was 7.87 ± 6.51 years in the antihistamine-effective group and 5.12 ± 3.87 years in the antihistamine-ineffective group (P = 0.254). Seasonal allergies were common in both groups (79.2% *vs*. 87.5%, P = 0.583). Interestingly, 32.7% of patients in the antihistamine-effective group exhibited allergies to specific allergens, whereas no such cases were observed in the antihistamine-ineffective group (P = 0.057). A family history of allergies was reported in 38.8% of patients in the antihistamine-effective group and 25.0% in the antihistamine-ineffective group (P = 0.454).

Overall, no statistically significant differences were identified in demographic or clinical characteristics between the two groups.

The comparison of serum vitamin levels and RNA expression between antihistamine-effective (n=49) and antihistamine-ineffective (n=8) groups revealed several notable findings. Serum vitamin D parameters, including total vitamin D (17.73 ± 6.81 *vs* 16.14 ± 7.10 ng/ml, P=0.546), vitamin D2 (1.52 ± 2.02 *vs* 1.24 ± 1.08 ng/ml, P=0.705), and vitamin D3 (16.21 ± 6.70 *vs* 14.91 ± 6.12 ng/ml, P=0.607), showed no significant differences between the groups (**Table 2**).

**Table 2:** Comparison of serum vitamin levels, blood mRNA and nasal swab mRNA expression between antihistamine-effective and antihistamine-ineffective allergic rhinitis patients.

Variables	Antihistamine-effective group (n=49)	Antihistamine-ineffective group(n=8)	P-value*
Vitamin D (20-100) ng/ml	17.73 ± 6.81	16.14 ± 7.10	0.546
Vitamin D2ng/ml	1.52 ± 2.02	1.24 ± 1.08	0.705
Vitamin D3ng/ml	16.21 ± 6.70	14.91 ± 6.12	0.607
Blood RNA			
eosinophil cationic protein (ECP) copies/µL	55.28 ± 74.90	107.63 ± 109.55	0.092
beta-pancreatin-like protein (TPSB)	1.57 ± 2.63	3.40 ± 5.79	0.140
cysteinyl leukotriene type 1 receptor (CYS1) copies/µL	165.93 ± 281.76	159.50 ± 135.11	0.950
CYS2 copies/µL	55.46 ± 99.08	51.36 ± 48.22	0.915
histamine type 1 receptor (H1R) copies/µL	1.22 ± 1.73	2.77 ± 3.27	**0.046**
H4R copies/µL	24.38 ± 45.53	21.65 ± 21.78	0.869
Nasal swab RNA			
ECP copies/µL	45.13 ± 45.90	63.51 ± 53.55	0.309
TPSB copies/µL	13.52 ± 21.79	38.68 ± 30.08	**0.006**
CYS1 copies/µL	31.49 ± 31.01	56.09 ± 55.69	0.072
CYS2 copies/µL	36.25 ± 42.76	50.54 ± 46.58	0.390
H1R copies/µL	32.82 ± 36.79	47.84 ± 40.07	0.295
H4R copies/µL	27.37 ± 28.69	38.22 ± 32.47	0.334

* and Bold value suggests p<0.05 for statistically different results.

In the analysis of blood mRNA, most parameters showed no statistically significant differences between the groups, except for H1R expression, which was significantly higher in the antihistamine-ineffective group (2.77 ± 3.27 *vs*. 1.22 ± 1.73 copies/µL, P = 0.046). Other blood RNA markers, including ECP, TPSB, CYS1, CYS2, and H4R, did not differ significantly between the groups. However, there was a notable trend toward higher ECP levels in the antihistamine-ineffective group (107.63 ± 109.55 *vs*. 55.28 ± 74.90 copies/µL, P = 0.092).

Analysis of nasal swab RNA revealed that TPSB expression was significantly higher in the antihistamine-ineffective group (38.68 ± 30.08 *vs*. 13.52 ± 21.79 copies/µL, P = 0.006). Additionally, CYS1 exhibited a trend toward higher expression in the antihistamine-ineffective group (56.09 ± 55.69 *vs*. 31.49 ± 31.01 copies/µL, P = 0.072), though this difference did not reach statistical significance. Other nasal swab RNA markers, including ECP, CYS2, H1R, and H4R, showed no significant differences between the two groups.

**Figure 1:**
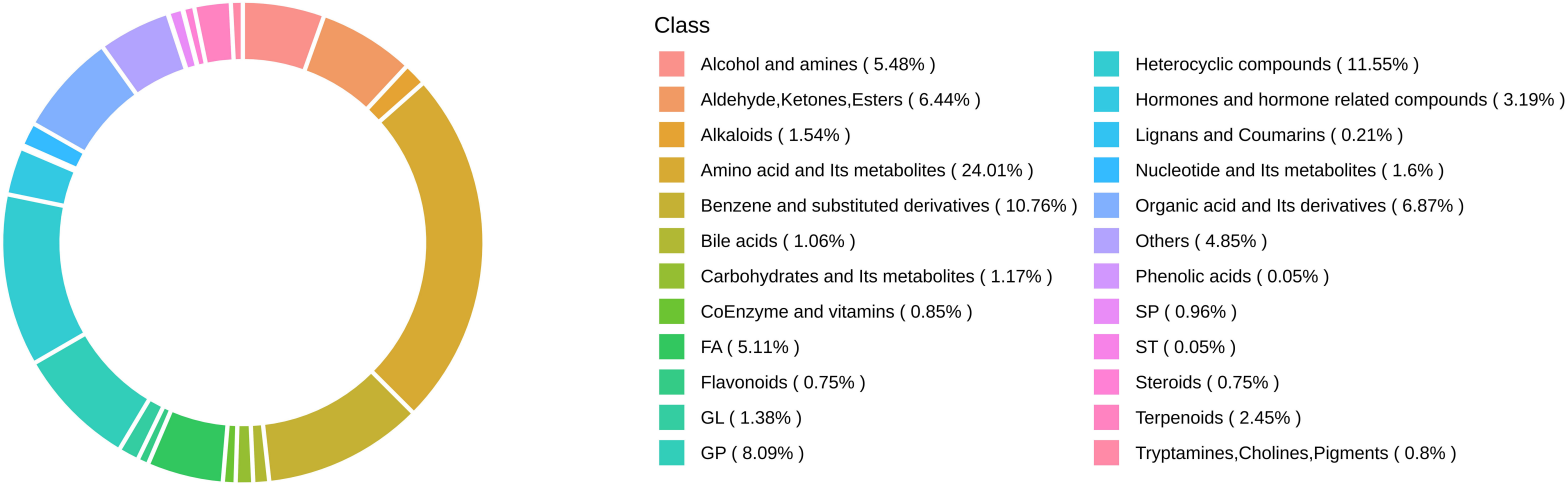
Metabolite class composition ring.

A total of 3,998 biochemicals were tested in this study. **Figure 1** illustrates the class composition of metabolites.

**Figure 2:**
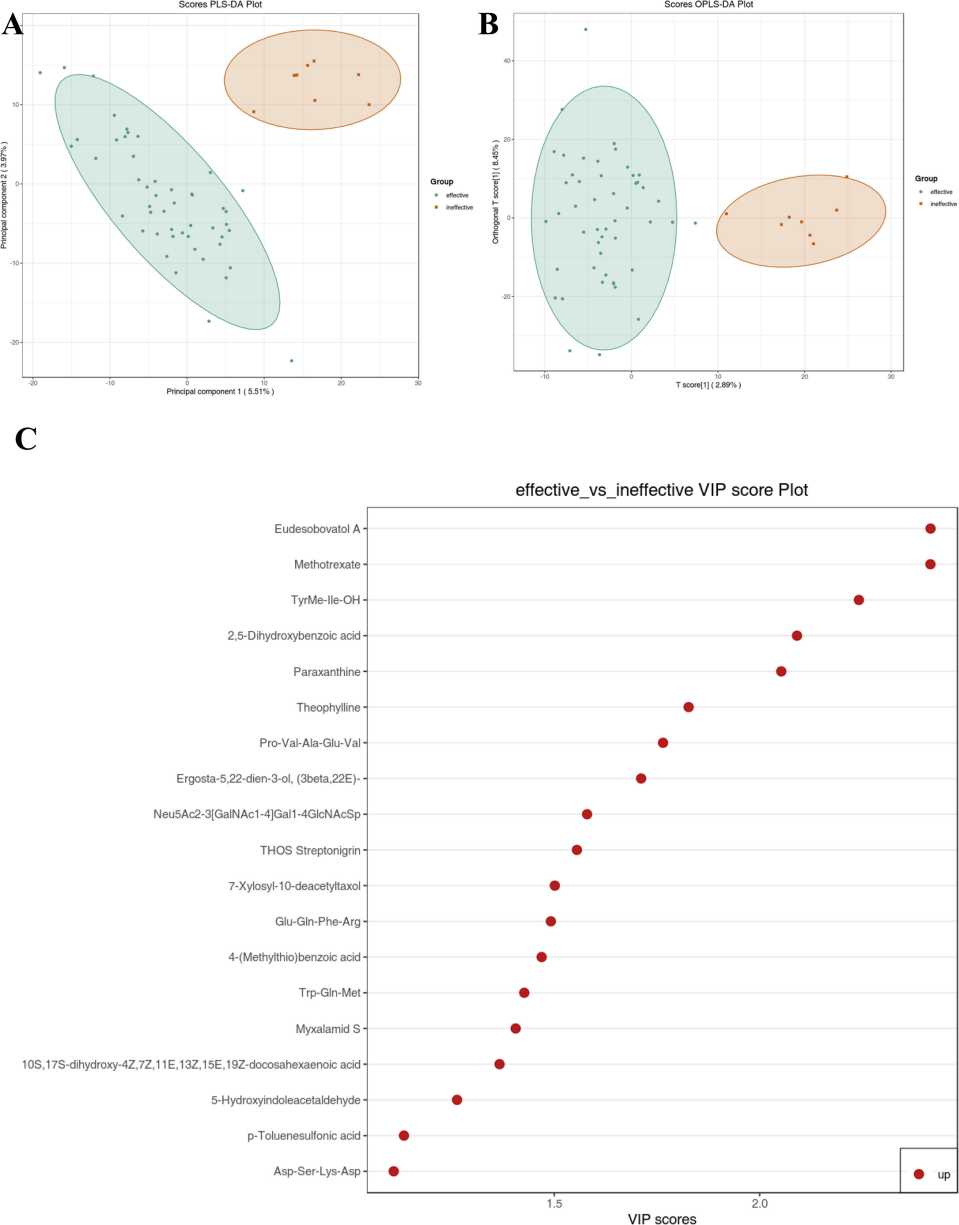
Metabolic data analysis of antihistamine-effective and antihistamine-ineffective AR patients was performed using multivariate statistical methods. **(A)** The score plot generated by Partial Least Squares Discriminant Analysis (PLS-DA) and **(B)** the score plot produced by Orthogonal Partial Least Squares Discriminant Analysis (OPLS-DA) visually demonstrate a clear separation in metabolomic profiles between antihistamine-effective and antihistamine-ineffective AR patients. **(C)** The VIP score plot, derived from the OPLS-DA model, identifies 19 metabolites that showed significant differences between the two groups. The colored boxes on the right side of the plot represent the relative concentration variations of the corresponding metabolites, with red indicating higher concentrations.

A comparison between antihistamine-effective and antihistamine-ineffective patients identified 19 metabolites that differed significantly, based on a cutoff of P < 0.05, fold change > 2.0, and Variable Importance in the Projection (VIP) > 1.0. In this study, an untargeted integrated metabolomics analysis using LC-MS was conducted to explore differences in metabolomic patterns between antihistamine-effective and antihistamine-ineffective AR patients. As shown in **Figure 2**, a clear separation between the two groups was achieved using both the PLS-DA (**Figure 2A**) and OPLS-DA (**Figure 2B**) models. In the PLS-DA model, all antihistamine-ineffective samples (red dots) were clustered on the right, while all antihistamine-effective samples (green dots) were clustered on the left. Similarly, in the OPLS-DA model, the two groups occupied the same respective positions (**Figure 2B**). Notably, no overlap was observed between the two groups in either model (**Figures 2A, B**). These results indicate significant differences in the metabolomic profiles between antihistamine-effective and antihistamine-ineffective patients.

**Figure 2C** presents the VIP plot, which was used to identify key metabolites contributing to the distinction between the drug-effective and drug-ineffective groups. The VIP plot, generated from the OPLS-DA model, highlights 19 significant compounds ranked according to their discriminatory power. Variables with a VIP score > 1 are considered highly influential. Among these, the principal metabolites contributing to the separation included Eudesobovatol A, Methotrexate, TyrMe-Ile-OH, 2,5-Dihydroxybenzoic acid, Paraxanthine, Theophylline, Pro-Val-Ala-Glu-Val, Ergosta-5,22-dien-3-ol (3beta,22E), Neu5Ac2-3(GalNAc1-4)Gal1-4GlcNAcSp, THOS Streptonigrin, 7-Xylosyl-10-deacetyltaxol, Glu-Gln-Phe-Arg, 4-(Methylthio)benzoic acid, Trp-Gln-Met, Myxalamid S, 10S,17S-dihydroxy-4Z,7Z,11E,13Z,15E,19Z-docosahexaenoic acid, 5-Hydroxyindoleacetaldehyde, p-Toluenesulfonic acid, and Asp-Ser-Lys-Asp. These metabolites were identified as the most influential contributors to the separation of the two groups.

**Figure 3:**
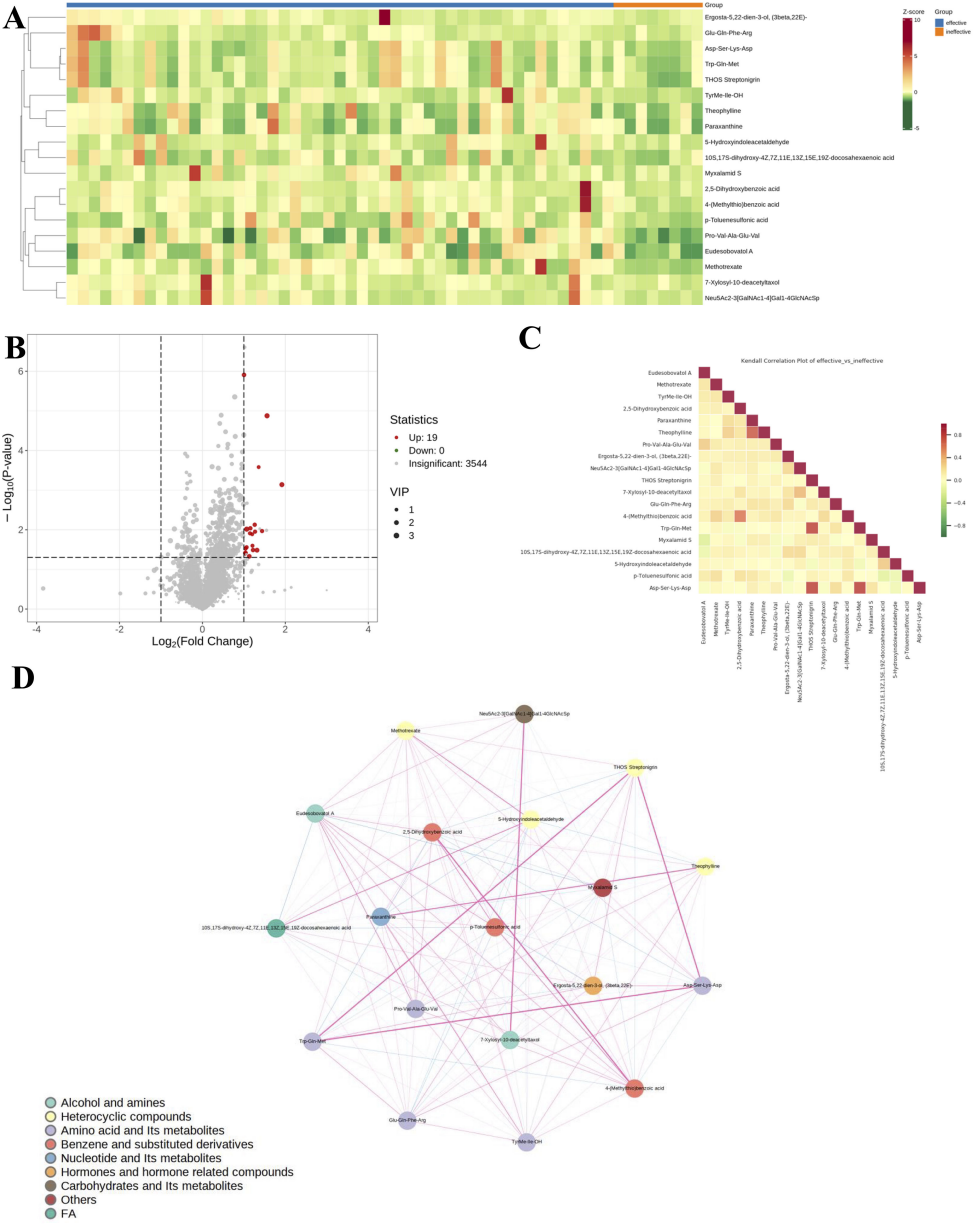
Variations in metabolite levels between antihistamine-effective and antihistamine-ineffective AR patients. **(A)** The heatmap displays the relative abundances of the top 19 metabolites and lipids, with red indicating increased abundance and green indicating decreased abundance. Columns represent samples, and rows represent metabolites. **(B)** The volcano plot highlights significantly different metabolites between the two groups, based on fold change and p-value. Red points indicate upregulated metabolites, while gray points represent non-significant ones. The horizontal axis shows the fold change (log scale), and the vertical axis indicates significance (p-value). Dot size reflects the VIP value. **(C)** The correlation heatmap shows Pearson correlation coefficients between metabolites, with red indicating strong positive correlations and green indicating strong negative correlations. Darker colors represent stronger correlations. **(D)** The metabolite correlation network diagram visualizes significantly different metabolites as dots, with dot size reflecting connectivity. Pink lines indicate positive correlations, blue lines indicate negative correlations, and line thickness represents the strength of the correlation.

Variations in metabolite levels between antihistamine-effective and antihistamine-ineffective AR patients are presented in **Figure 3**. Specifically, a heatmap (**Figure 3A**) illustrates the relative abundances of the top 19 significant metabolites, with color intensity representing differences between the two groups. Additionally, a volcano plot analysis (**Figure 3B**) was performed by integrating fold change values (>2.0, antihistamine-effective/antihistamine-ineffective) and FDR-corrected p-values (<0.05) as cutoff criteria, highlighting the metabolites that significantly contributed to the distinction between the groups. A total of 19 metabolites were upregulated in the antihistamine-effective group compared to the antihistamine-ineffective group. **Figure 3C** displays the differential metabolite correlation heatmap, with the legend on the right indicating the relationship between correlation coefficients and colors. Lastly, **Figure 3D** presents the differential metabolite correlation network diagram, where the dots represent significantly different metabolites, and the size of each dot reflects its degree of connectivity within the network.

**Figure 4:**
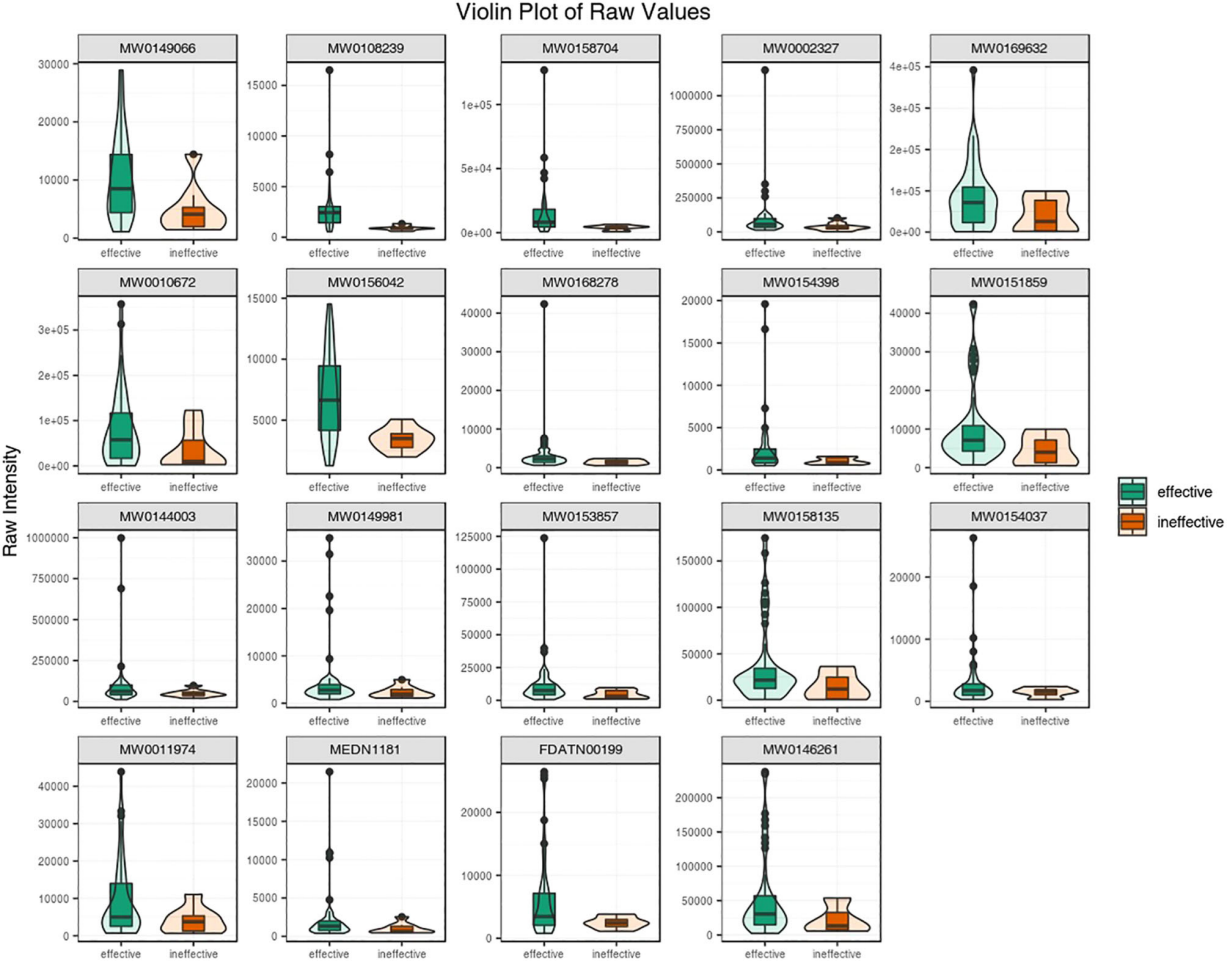
A violin plot combines a box plot and a density plot to display data distributions and probability densities. The central box represents the interquartile range, the thin black line indicates the 95% confidence interval, and the black horizontal line marks the median. The outer shape reflects the data distribution density. The horizontal axis represents sample groups, and the vertical axis shows the relative content of differential metabolites (raw peak area). P-values were calculated using the Student’s t-test.

Subsequently, the top 19 statistically significant metabolites are presented in **Figure 4**. Notably, all 19 metabolites exhibited increased concentrations in antihistamine-effective AR patients compared to antihistamine-ineffective AR patients. To further evaluate the differences between the two groups, a violin plot analysis of peak intensities for the top 50 significant compounds was performed, with the results also shown in **Figure 4**. Details of the metabolites corresponding to the codes in **Figure 4** are provided in **Supplementary File 1**.

**Figure 5:**
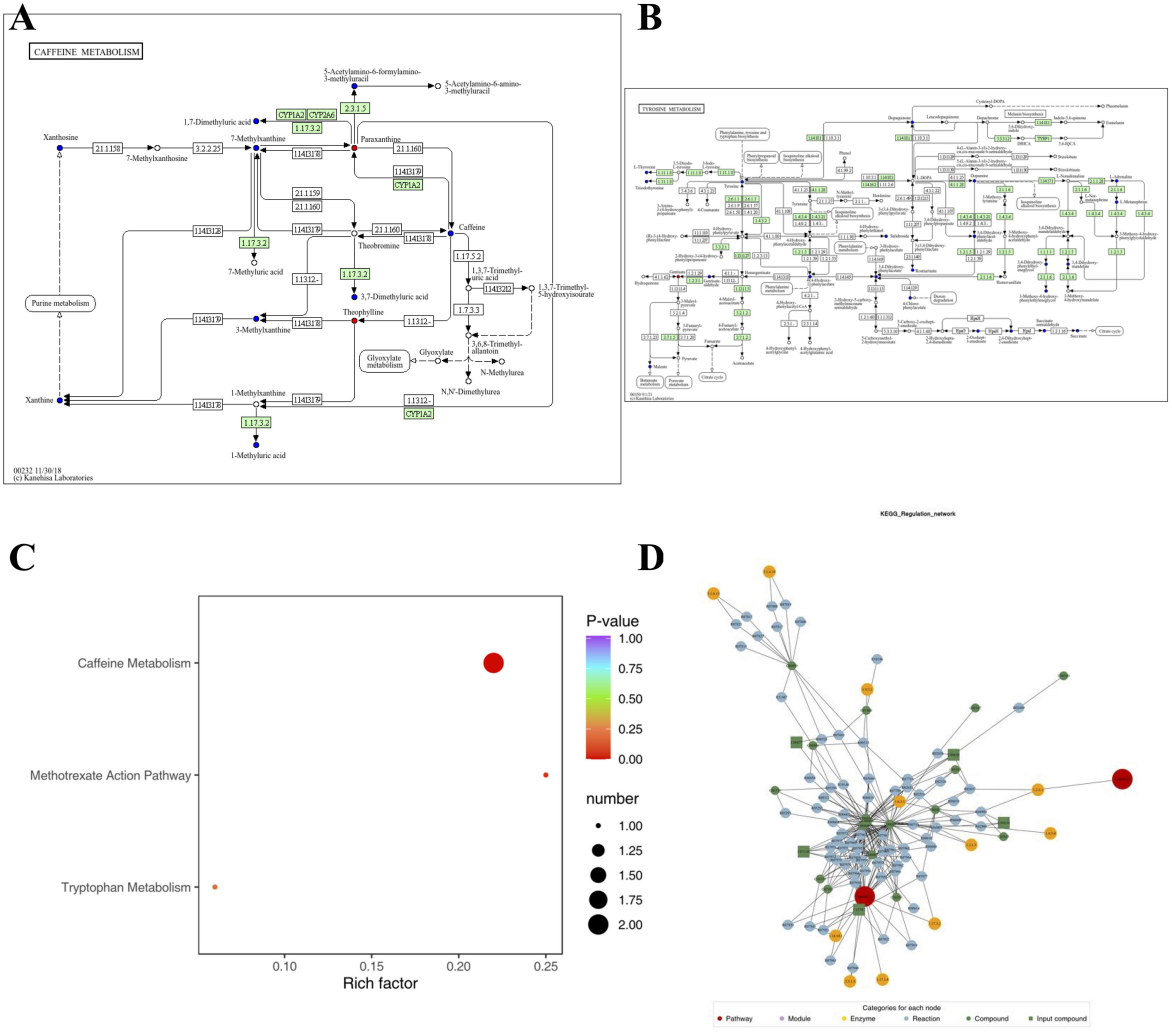
The pathways associated with each identified compound were determined using the Kyoto Encyclopedia of Genes and Genomes (KEGG) pathway database. **(A, B)** KEGG pathway maps of differential metabolites: red indicates significantly upregulated metabolites, green indicates significantly downregulated metabolites, blue indicates metabolites detected without significant changes, and orange represents pathways containing both upregulated and downregulated metabolites. Only two pathway maps are shown as examples; additional details are provided in **Supplementary File 2**. **(C)** KEGG enrichment analysis of differential metabolites: dot size represents the number of significantly enriched metabolites in each pathway, with the top three pathways (based on p-value) displayed. **(D)** Network plot based on the KEGG database for the corresponding species.

**Figure 5** highlights the metabolic pathways associated with the identified compounds, as determined using the Kyoto Encyclopedia of Genes and Genomes (KEGG) pathway database. The differentially expressed compounds between antihistamine-effective and antihistamine-ineffective AR patients are primarily involved in the Caffeine Metabolism and Tryptophan Metabolism pathways (**Figures 5A, B**), with additional pathways detailed in **Supplementary File 2**. These metabolic pathways provide insights into the potential causes of phenotypic differences among the study subjects. The KEGG enrichment analysis of differential metabolites is shown in **Figure 5C**, including pathways such as Caffeine Metabolism, Methotrexate Action Pathway, and Tryptophan Metabolism. Furthermore, pathway searches and regulatory interaction network analyses were conducted based on the KEGG database for the corresponding species, and the results are presented as a network plot in **Figure 5D**.

**Figure 6:**
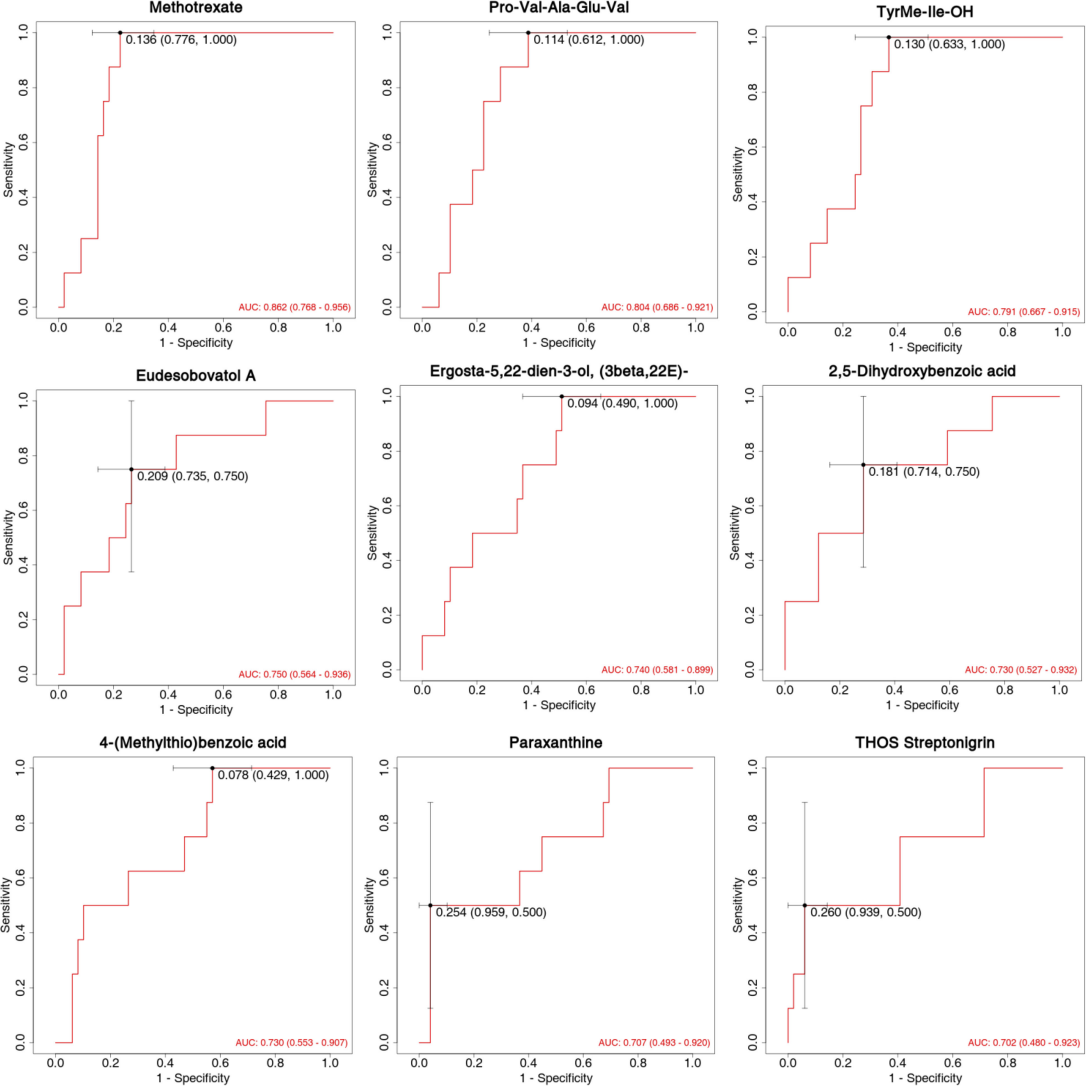
ROC curve analysis of statistically significant compounds (AUC>0.70).

The diagnostic performance of differentially expressed metabolites as potential biomarkers was evaluated using ROC curves, focusing on sensitivity and specificity (**Figure 6**). The area under the ROC curve (AUC) was used to assess the accuracy and efficiency of distinguishing antihistamine-effective from antihistamine-ineffective AR patients. Among the differentially expressed metabolites, 9 showed the highest AUC values (AUC > 0.70). Notably, Methotrexate (AUC = 0.862, 95% CI: 0.768–0.956), Pro-Val-Ala-Glu-Val (AUC = 0.804, 95% CI: 0.686–0.921), and TyrMe-Ile-OH (AUC = 0.791, 95% CI: 0.667–0.915) demonstrated AUC values around 0.8, indicating strong discriminative ability between the two groups. Other compounds, including Eudesobovatol A, Ergosta-5,22-dien-3-ol (3beta,22E), 2,5-Dihydroxybenzoic acid, 4-(Methylthio)benzoic acid, Paraxanthine, and THOS Streptonigrin, exhibited AUC values around or above 0.70, suggesting moderate discriminatory capability (**Figure 6**).

## Discussion

This study provides a comprehensive analysis of metabolomic profiles in antihistamine-effective and antihistamine-ineffective allergic rhinitis patients, uncovering key findings that may inform personalized treatment strategies. Our results identified distinct metabolomic signatures linked to treatment response, emphasizing the involvement of specific metabolic pathways and potential biomarkers.

The most notable finding of this study was the identification of 19 differential metabolites between the antihistamine-effective and antihistamine-ineffective groups. Among these, nine metabolites exhibited strong diagnostic potential (AUC > 0.70), with Methotrexate, Pro-Val-Ala-Glu-Val, and TyrMe-Ile-OH demonstrating the highest discriminatory power. These results suggest that metabolomic profiling could be a valuable tool for predicting treatment responses in AR patients. Although nine metabolites showed high diagnostic potential, we did not assess their clinical utility, including detection cost and feasibility. Future studies should evaluate these aspects to facilitate translation into clinical practice. We did not conduct a detailed analysis of the relationships between metabolite levels and specific clinical symptoms (e.g., nasal congestion, rhinorrhea, sneezing). Further research should investigate these associations to enhance the clinical relevance of the identified biomarkers.

Interestingly, there are no prior reports linking methotrexate to allergic disease metabolomics. Considering that all patients were excluded from taking immunosuppressive antihistamines, it is possible that the detected methotrexate-related signals represent intermediates or analogs in metabolic processes rather than the drug itself.

Tyrosine has been previously implicated in allergic rhinitis in several studies. For example, serum TAM receptor tyrosine kinase levels have been suggested as potential indicators of disease severity and predictors of sublingual immunotherapy (SLIT) responsiveness in AR patients (12). Additionally, polymorphisms in the PTPN22 (protein tyrosine phosphatase non-receptor 22) and CTLA-4 (cytotoxic T lymphocyte-associated antigen 4) genes have been strongly associated with asthma prevalence and morbidity (13). In our study, higher levels of TyrMe-Ile-OH were observed in the antihistamine-effective group, potentially indicating that lower levels of TyrMe-Ile-OH may be associated with greater difficulty in identifying effective therapeutic agents. This finding aligns with previous research, further supporting its relevance.

The significantly higher expression of H1R in the blood of antihistamine-ineffective patients (P = 0.046) is particularly noteworthy. H1R plays a critical role in allergic reactions, as histamine exerts its effects by binding to H1 receptors. In allergic rhinitis, antihistamines alleviate symptoms by blocking H1 receptors. However, prolonged use of antihistamines may lead to compensatory upregulation of H1 receptors, characterized by increased H1R mRNA and protein expression, to counteract the inhibitory effects of antihistamines. This compensatory mechanism has been proposed as a potential cause of antihistamine resistance (14). Our findings support the hypothesis that H1R expression levels may significantly influence treatment response, particularly in relation to antihistamine therapy.

The significantly elevated TPSB expression in nasal swabs of antihistamine-ineffective patients (P = 0.006) suggests a more pronounced local inflammatory response in these individuals. The TPSB gene encodes tryptase-like protease B (Tryptase Beta), a protein specifically secreted by mast cells that plays a key role in allergic and inflammatory responses (15). Mast cells are central to the pathophysiology of allergic rhinitis. Upon allergen stimulation, mast cells degranulate, releasing inflammatory mediators such as histamine and tryptase, which trigger the symptoms of allergic rhinitis. In antihistamine-resistant patients, the diminished efficacy of antihistamines in controlling symptoms may result in further mast cell activation and the subsequent release of additional inflammatory mediators, including tryptase. This process likely contributes to the observed increase in TPSB RNA expression. Elevated TPSB RNA levels may serve as a biomarker for heightened inflammatory activity and more severe disease in antihistamine-resistant patients.

Pathway analysis revealed significant enrichment in the caffeine metabolism and tryptophan metabolism pathways. The involvement of caffeine metabolism is particularly intriguing, as it points to a potential role of methylxanthines in influencing treatment response (16). This finding is consistent with previous studies demonstrating the anti-inflammatory effects of methylxanthines in allergic conditions (17). The enrichment of the tryptophan metabolism pathway suggests that this essential amino acid plays a critical role in treatment response (18). Tryptophan metabolites are known to modulate immune responses through various mechanisms, including the regulation of T cell activity and the production of inflammatory mediators (19, 20). The differential expression of tryptophan-related metabolites between the antihistamine-effective and -ineffective groups highlights their potential as novel therapeutic targets for allergic rhinitis treatment.

Several limitations of our study should be acknowledged. First, the relatively small sample size, especially in the antihistamine-ineffective group, may reduce statistical power and increase the risk of random findings, thus restricting the reliability and generalizability of our results. Future studies with larger, multicenter cohorts are warranted to validate and expand upon these findings. Second, as an observational study, we can only describe associations between metabolomic profiles and antihistamine response, but cannot determine whether the observed metabolic changes are causative or consequential. Mechanistic and longitudinal studies are needed to clarify these relationships.

Additionally, although we excluded patients recently using immunosuppressants or antibiotics, and all participants were required to fast prior to blood sample collection to minimize the acute effects of dietary intake, other potential confounding factors such as long-term dietary habits, lifestyle, and comorbidities were not systematically controlled for, which may have influenced the metabolomic results. While we identified 19 significantly different metabolites, we did not perform functional studies to elucidate their roles in AR pathophysiology or antihistamine response. Future research should combine metabolomic analysis with *in vitro* or animal model experiments to validate the biological functions and mechanisms of these metabolites. We did not differentiate between different types or generations of antihistamines, which may have distinct metabolic and efficacy profiles. Future studies with larger sample sizes should stratify patients by antihistamine type to refine metabolomic associations.

In conclusion, this study demonstrates that metabolomic profiling can effectively differentiate between antihistamine-effective and antihistamine-ineffective allergic rhinitis patients. The identification of specific metabolic signatures, particularly the nine metabolites with high diagnostic potential, highlights promising biomarkers for predicting treatment response. Alongside the findings of differential H1R and TPSB expression, these results provide valuable insights into the biological mechanisms underlying treatment response variability in allergic rhinitis. This work marks a significant step toward personalized medicine in the management of allergic rhinitis, paving the way for more targeted and effective therapeutic strategies. However, further validation in larger cohorts and the development of clinical applications will be essential to fully translate these findings into practical diagnostic and treatment tools.

## Data Availability

The original contributions presented in the study are included in the article/**Supplementary Material**. Further inquiries can be directed to the corresponding authors.
