# Supplementary material for: Metabolomic insights into variable antihistamine responses in allergic rhinitis: unveiling biomarkers for precision treatment
Source: Front Immunol. 2025 Jun 17;16:1565972. doi: 10.3389/fimmu.2025.1565972 (PMC12209198; doi:10.3389/fimmu.2025.1565972)
Supplement: Supplementary file 1 [file DataSheet1.zip › Supplementary file 2/ko04976.html]

KEGG PATHWAY: Bile secretion - Homo sapiens (human)


# Bile secretion - Homo sapiens (human)


[
Pathway menu
|
Organism menu
|
Pathway entry
|
Show description
|
Download
|
Help
]

Bile is a vital secretion, essential for digestion and absorption of fats and fat-soluble vitamins in the small intestine. Moreover, bile is an important route of elimination for excess cholesterol and many waste product, bilirubin, drugs and toxic compounds. Bile secretion depends on the function of membrane transport systems in hepatocytes and cholangiocytes and on the structural and functional integrity of the biliary tree. The hepatocytes generate the so-called primary bile in their canaliculi. Cholangiocytes modify the canalicular bile by secretory and reabsorptive processes as bile passes through the bile ducts. The main solutes in bile are bile acids, which stimulate bile secretion osmotically, as well as facilitate the intestinal absorption of dietary lipids by their detergent properties. Bile acids are also important signalling molecules. Through the activation of nuclear receptors, they regulate their own synthesis and transport rates.


##### Option

Scale:


100%

Image resolution:


High

##### Background color

Organism

Split cells that has orgs.
  


Exclude cells that has no orgs.

##### Search

##### ID search

##### Color


KGML

Image (png) file 1x

Image (png) file 2x
