# Supplementary material for: Metabolomic insights into variable antihistamine responses in allergic rhinitis: unveiling biomarkers for precision treatment
Source: Front Immunol. 2025 Jun 17;16:1565972. doi: 10.3389/fimmu.2025.1565972 (PMC12209198; doi:10.3389/fimmu.2025.1565972)
Supplement: Supplementary file 1 [file DataSheet1.zip › Supplementary file 2/ko00232.html]

KEGG PATHWAY: Caffeine metabolism - Homo sapiens (human)


# Caffeine metabolism - Homo sapiens (human)


[
Pathway menu
|
Organism menu
|
Pathway entry
|
Download
|
Help
]


##### Option

Scale:


100%

Image resolution:


High

##### Background color

Organism

Split cells that has orgs.
  


Exclude cells that has no orgs.

##### Search

##### ID search

##### Color

##### Module

Complete only

Including 1 block missing

Including any incomplete

- Pathway modules
  - Xenobiotics biodegradation
    - Aromatics degradation
      - M00915
        Caffeine degradation


KGML

Image (png) file 1x

Image (png) file 2x
