# Supplementary material for: Metabolomic insights into variable antihistamine responses in allergic rhinitis: unveiling biomarkers for precision treatment
Source: Front Immunol. 2025 Jun 17;16:1565972. doi: 10.3389/fimmu.2025.1565972 (PMC12209198; doi:10.3389/fimmu.2025.1565972)
Supplement: Supplementary file 1 [file DataSheet1.zip › Supplementary file 2/ko01100.html]

KEGG PATHWAY: Metabolic pathways - Homo sapiens (human)


# Metabolic pathways - Homo sapiens (human)


[
Pathway menu
|
Pathway entry
|
Download
|
Help
]


##### Option

Scale:


30%

Image resolution:


High

Coloring:


Yes

No

Link:


Normal

Module

##### Background color

Organism

Split cells that has orgs.
  


Exclude cells that has no orgs.

##### Search

##### ID search

##### Color

##### Module

Complete only

Including 1 block missing

Including any incomplete

- Pathway modules
  - Carbohydrate metabolism
    - Central carbohydrate metabolism
      - M00001
        Glycolysis (Embden-Meyerhof pathway)
      - M00002
        Glycolysis, core module involving three-carbon compounds
      - M00003
        Gluconeogenesis
      - M00307
        Pyruvate oxidation
      - M00009
        Citrate cycle (TCA cycle, Krebs cycle)
      - M00010
        Citrate cycle, first carbon oxidation
      - M00011
        Citrate cycle, second carbon oxidation
      - M00004
        Pentose phosphate pathway (Pentose phosphate cycle)
      - M00006
        Pentose phosphate pathway, oxidative phase
      - M00007
        Pentose phosphate pathway, non-oxidative phase
      - M00580
        Pentose phosphate pathway, archaea
      - M00005
        PRPP biosynthesis
      - M00008
        Entner-Doudoroff pathway
      - M00308
        Semi-phosphorylative Entner-Doudoroff pathway
      - M00633
        Semi-phosphorylative Entner-Doudoroff pathway
      - M00309
        Non-phosphorylative Entner-Doudoroff pathway
    - Other carbohydrate metabolism
      - M00014
        Glucuronate pathway (uronate pathway)
      - M00630
        D-Galacturonate degradation (fungi)
      - M00631
        D-Galacturonate degradation (bacteria)
      - M00061
        D-Glucuronate degradation
      - M00081
        Pectin degradation
      - M00632
        Galactose degradation, Leloir pathway
      - M00552
        D-galactonate degradation, De Ley-Doudoroff pathway
      - M00129
        Ascorbate biosynthesis, animals
      - M00114
        Ascorbate biosynthesis, plants
      - M00550
        Ascorbate degradation
      - M00854
        Glycogen biosynthesis
      - M00855
        Glycogen degradation
      - M00565
        Trehalose biosynthesis
      - M00549
        Nucleotide sugar biosynthesis
      - M00554
        Nucleotide sugar biosynthesis
      - M00892
        UDP-N-acetyl-D-glucosamine biosynthesis, eukaryotes
      - M00761
        Undecaprenylphosphate alpha-L-Ara4N biosynthesis
      - M00012
        Glyoxylate cycle
      - M00373
        Ethylmalonyl pathway
      - M00740
        Methylaspartate cycle
      - M00532
        Photorespiration
      - M00013
        Malonate semialdehyde pathway
      - M00741
        Propanoyl-CoA metabolism
      - M00968
        Pentose bisphosphate pathway (nucleoside degradation), archaea
      - M00130
        Inositol phosphate metabolism
      - M00131
        Inositol phosphate metabolism
      - M00132
        Inositol phosphate metabolism
  - Energy metabolism
    - Carbon fixation
      - M00165
        Reductive pentose phosphate cycle (Calvin cycle)
      - M00168
        CAM (Crassulacean acid metabolism), dark
      - M00169
        CAM (Crassulacean acid metabolism), light
      - M00172
        C4-dicarboxylic acid cycle, NADP - malic enzyme type
      - M00171
        C4-dicarboxylic acid cycle, NAD - malic enzyme type
      - M00170
        C4-dicarboxylic acid cycle, phosphoenolpyruvate carboxykinase type
      - M00173
        Reductive citrate cycle (Arnon-Buchanan cycle)
      - M00376
        3-Hydroxypropionate bi-cycle
      - M00375
        Hydroxypropionate-hydroxybutylate cycle
      - M00374
        Dicarboxylate-hydroxybutyrate cycle
      - M00377
        Reductive acetyl-CoA pathway (Wood-Ljungdahl pathway)
      - M00579
        Phosphate acetyltransferase-acetate kinase pathway
      - M00620
        Incomplete reductive citrate cycle
    - Methane metabolism
      - M00567
        Methanogenesis
      - M00357
        Methanogenesis
      - M00356
        Methanogenesis
      - M00563
        Methanogenesis
      - M00358
        Coenzyme M biosynthesis
      - M00608
        2-Oxocarboxylic acid chain extension
      - M00174
        Methane oxidation, methanotroph
      - M00346
        Formaldehyde assimilation, serine pathway
      - M00345
        Formaldehyde assimilation, ribulose monophosphate pathway
      - M00344
        Formaldehyde assimilation, xylulose monophosphate pathway
      - M00378
        F420 biosynthesis, archaea
      - M00935
        Methanofuran biosynthesis
      - M00422
        Acetyl-CoA pathway
    - Nitrogen metabolism
      - M00175
        Nitrogen fixation
      - M00531
        Assimilatory nitrate reduction
      - M00530
        Dissimilatory nitrate reduction
      - M00529
        Denitrification
      - M00528
        Nitrification
      - M00804
        Complete nitrification, comammox
      - M00973
        Anammox
    - Sulfur metabolism
      - M00176
        Assimilatory sulfate reduction
      - M00596
        Dissimilatory sulfate reduction
      - M00595
        Thiosulfate oxidation by SOX complex
    - Photosynthesis
      - M00161
        Photosystem II
      - M00163
        Photosystem I
    - ATP synthesis
      - M00144
        NADH:quinone oxidoreductase, prokaryotes
      - M00145
        NAD(P)H:quinone oxidoreductase, chloroplasts and cyanobacteria
      - M00142
        NADH:ubiquinone oxidoreductase, mitochondria
      - M00143
        NADH dehydrogenase (ubiquinone) Fe-S protein/flavoprotein complex, mitochondria
      - M00146
        NADH dehydrogenase (ubiquinone) 1 alpha subcomplex
      - M00147
        NADH dehydrogenase (ubiquinone) 1 beta subcomplex
      - M00149
        Succinate dehydrogenase, prokaryotes
      - M00150
        Fumarate reductase, prokaryotes
      - M00148
        Succinate dehydrogenase (ubiquinone)
      - M00162
        Cytochrome b6f complex
      - M00151
        Cytochrome bc1 complex respiratory unit
      - M00152
        Cytochrome bc1 complex
      - M00154
        Cytochrome c oxidase
      - M00155
        Cytochrome c oxidase, prokaryotes
      - M00153
        Cytochrome bd ubiquinol oxidase
      - M00417
        Cytochrome o ubiquinol oxidase
      - M00416
        Cytochrome aa3-600 menaquinol oxidase
      - M00156
        Cytochrome c oxidase, cbb3-type
      - M00157
        F-type ATPase, prokaryotes and chloroplasts
      - M00158
        F-type ATPase, eukaryotes
      - M00160
        V-type ATPase, eukaryotes
  - Lipid metabolism
    - Fatty acid metabolism
      - M00082
        Fatty acid biosynthesis, initiation
      - M00083
        Fatty acid biosynthesis, elongation
      - M00873
        Fatty acid biosynthesis in mitochondria, animals
      - M00874
        Fatty acid biosynthesis in mitochondria, fungi
      - M00085
        Fatty acid elongation in mitochondria
      - M00415
        Fatty acid elongation in endoplasmic reticulum
      - M00086
        beta-Oxidation, acyl-CoA synthesis
      - M00087
        beta-Oxidation
      - M00861
        beta-Oxidation, peroxisome, VLCFA
    - Sterol biosynthesis
      - M00101
        Cholesterol biosynthesis
      - M00102
        Ergocalciferol biosynthesis
      - M00917
        Phytosterol biosynthesis
      - M00103
        Cholecalciferol biosynthesis
      - M00104
        Bile acid biosynthesis
      - M00106
        Conjugated bile acid biosynthesis
      - M00862
        beta-Oxidation, peroxisome
      - M00107
        Steroid hormone biosynthesis
      - M00108
        C21-Steroid hormone biosynthesis
      - M00109
        C21-Steroid hormone biosynthesis
      - M00110
        C19/C18-Steroid hormone biosynthesis
      - M00976
        C19-Steroid hormone biosynthesis
      - M00977
        C19-Steroid hormone biosynthesis (androgen backdoor pathway)
    - Lipid metabolism
      - M00088
        Ketone body biosynthesis
      - M00089
        Triacylglycerol biosynthesis
      - M00098
        Acylglycerol degradation
      - M00090
        Phosphatidylcholine (PC) biosynthesis
      - M00091
        Phosphatidylcholine (PC) biosynthesis
      - M00092
        Phosphatidylethanolamine (PE) biosynthesis
      - M00093
        Phosphatidylethanolamine (PE) biosynthesis
      - M00094
        Ceramide biosynthesis
      - M00066
        Lactosylceramide biosynthesis
      - M00067
        Sulfoglycolipids biosynthesis
      - M00099
        Sphingosine biosynthesis
      - M00100
        Sphingosine degradation
      - M00113
        Jasmonic acid biosynthesis
  - Nucleotide metabolism
    - Purine metabolism
      - M00048
        De novo purine biosynthesis
      - M00049
        Adenine ribonucleotide biosynthesis
      - M00050
        Guanine ribonucleotide biosynthesis
      - M00053
        Deoxyribonucleotide biosynthesis
      - M00958
        Adenine ribonucleotide degradation
      - M00959
        Guanine ribonucleotide degradation
      - M00546
        Purine degradation
    - Pyrimidine metabolism
      - M00051
        De novo pyrimidine biosynthesis
      - M00052
        Pyrimidine ribonucleotide biosynthesis
      - M00938
        Pyrimidine deoxyribonucleotide biosynthesis
      - M00046
        Pyrimidine degradation
      - M00939
        Pyrimidine degradation
  - Amino acid metabolism
    - Serine and threonine metabolism
      - M00020
        Serine biosynthesis
      - M00018
        Threonine biosynthesis
      - M00621
        Glycine cleavage system
      - M00555
        Betaine biosynthesis
      - M00974
        Betaine metabolism, animals
      - M00975
        Betaine degradation, bacteria
      - M00033
        Ectoine biosynthesis
      - M00919
        Ectoine degradation
    - Cysteine and methionine metabolism
      - M00021
        Cysteine biosynthesis
      - M00338
        Cysteine biosynthesis
      - M00609
        Cysteine biosynthesis
      - M00017
        Methionine biosynthesis
      - M00034
        Methionine salvage pathway
      - M00035
        Methionine degradation
      - M00368
        Ethylene biosynthesis
    - Branched-chain amino acid metabolism
      - M00019
        Valine/isoleucine biosynthesis
      - M00535
        Isoleucine biosynthesis
      - M00570
        Isoleucine biosynthesis
      - M00432
        Leucine biosynthesis
      - M00036
        Leucine degradation
    - Lysine metabolism
      - M00016
        Lysine biosynthesis, succinyl-DAP pathway
      - M00525
        Lysine biosynthesis, acetyl-DAP pathway
      - M00526
        Lysine biosynthesis, DAP dehydrogenase pathway
      - M00527
        Lysine biosynthesis, DAP aminotransferase pathway
      - M00030
        Lysine biosynthesis, AAA pathway
      - M00433
        Lysine biosynthesis
      - M00031
        Lysine biosynthesis, mediated by LysW
      - M00032
        Lysine degradation
      - M00956
        Lysine degradation, bacteria
      - M00957
        Lysine degradation, bacteria
      - M00960
        Lysine degradation, bacteria
    - Arginine and proline metabolism
      - M00028
        Ornithine biosynthesis
      - M00763
        Ornithine biosynthesis, mediated by LysW
      - M00844
        Arginine biosynthesis
      - M00845
        Arginine biosynthesis
      - M00029
        Urea cycle
      - M00015
        Proline biosynthesis
      - M00970
        Proline degradation
      - M00972
        Proline metabolism
      - M00047
        Creatine pathway
      - M00879
        Arginine succinyltransferase pathway
    - Polyamine biosynthesis
      - M00133
        Polyamine biosynthesis
      - M00134
        Polyamine biosynthesis
      - M00135
        GABA biosynthesis, eukaryotes
      - M00136
        GABA biosynthesis, prokaryotes
    - Histidine metabolism
      - M00026
        Histidine biosynthesis
      - M00045
        Histidine degradation
    - Aromatic amino acid metabolism
      - M00022
        Shikimate pathway
      - M00023
        Tryptophan biosynthesis
      - M00024
        Phenylalanine biosynthesis
      - M00910
        Phenylalanine biosynthesis
      - M00025
        Tyrosine biosynthesis
      - M00040
        Tyrosine biosynthesis
      - M00042
        Catecholamine biosynthesis
      - M00043
        Thyroid hormone biosynthesis
      - M00044
        Tyrosine degradation
      - M00533
        Homoprotocatechuate degradation
      - M00037
        Melatonin biosynthesis, animals
      - M00936
        Melatonin biosynthesis, plants
      - M00038
        Tryptophan metabolism
    - Other amino acid metabolism
      - M00027
        GABA (gamma-Aminobutyrate) shunt
      - M00369
        Cyanogenic glycoside biosynthesis
      - M00118
        Glutathione biosynthesis
      - M00947
        D-Arginine racemization
      - M00948
        Hydroxyproline degradation
      - M00949
        Staphylopine biosynthesis
  - Glycan metabolism
    - Glycan biosynthesis
      - M00055
        N-glycan precursor biosynthesis
      - M00072
        N-glycosylation by oligosaccharyltransferase
      - M00073
        N-glycan precursor trimming
      - M00074
        N-glycan biosynthesis, high-mannose type
      - M00075
        N-glycan biosynthesis, complex type
      - M00056
        O-glycan biosynthesis, mucin type core
      - M00872
        O-glycan biosynthesis, mannose type (core M3)
      - M00065
        GPI-anchor biosynthesis, core oligosaccharide
      - M00070
        Glycosphingolipid biosynthesis, lacto-series
      - M00071
        Glycosphingolipid biosynthesis, neolacto-series
      - M00068
        Glycosphingolipid biosynthesis, globo-series
      - M00069
        Glycosphingolipid biosynthesis, ganglio series
    - Glycosaminoglycan metabolism
      - M00057
        Glycosaminoglycan biosynthesis, linkage tetrasaccharide
      - M00058
        Glycosaminoglycan biosynthesis, chondroitin sulfate backbone
      - M00059
        Glycosaminoglycan biosynthesis, heparan sulfate backbone
      - M00076
        Dermatan sulfate degradation
      - M00077
        Chondroitin sulfate degradation
      - M00078
        Heparan sulfate degradation
      - M00079
        Keratan sulfate degradation
    - Lipopolysaccharide metabolism
      - M00060
        KDO2-lipid A biosynthesis, Raetz pathway, LpxL-LpxM type
      - M00866
        KDO2-lipid A biosynthesis, Raetz pathway, non-LpxL-LpxM type
      - M00867
        KDO2-lipid A modification pathway
      - M00063
        CMP-KDO biosynthesis
      - M00064
        ADP-L-glycero-D-manno-heptose biosynthesis
      - M00922
        CMP-Neu5Ac biosynthesis
      - M00923
        UDP-L-FucNAm biosynthesis
  - Metabolism of cofactors and vitamins
    - Cofactor and vitamin metabolism
      - M00127
        Thiamine biosynthesis, prokaryotes
      - M00895
        Thiamine biosynthesis, prokaryotes
      - M00896
        Thiamine biosynthesis, archaea
      - M00897
        Thiamine biosynthesis, plants
      - M00898
        Thiamine biosynthesis
      - M00899
        Thiamine salvage pathway
      - M00125
        Riboflavin biosynthesis, plants and bacteria
      - M00911
        Riboflavin biosynthesis, fungi
      - M00124
        Pyridoxal-P biosynthesis
      - M00916
        Pyridoxal-P biosynthesis
      - M00115
        NAD biosynthesis
      - M00912
        NAD biosynthesis
      - M00810
        Nicotine degradation, pyridine pathway
      - M00811
        Nicotine degradation, pyrrolidine pathway
      - M00622
        Nicotinate degradation
      - M00119
        Pantothenate biosynthesis
      - M00913
        Pantothenate biosynthesis
      - M00120
        Coenzyme A biosynthesis
      - M00914
        Coenzyme A biosynthesis, archaea
      - M00572
        Pimeloyl-ACP biosynthesis, BioC-BioH pathway
      - M00123
        Biotin biosynthesis
      - M00950
        Biotin biosynthesis, BioU pathway
      - M00573
        Biotin biosynthesis, BioI pathway
      - M00577
        Biotin biosynthesis, BioW pathway
      - M00881
        Lipoic acid biosynthesis, plants and bacteria
      - M00882
        Lipoic acid biosynthesis, eukaryotes
      - M00883
        Lipoic acid biosynthesis, animals and bacteria
      - M00884
        Lipoic acid biosynthesis
      - M00126
        Tetrahydrofolate biosynthesis
      - M00840
        Tetrahydrofolate biosynthesis, mediated by ribA and trpF
      - M00841
        Tetrahydrofolate biosynthesis, mediated by PTPS
      - M00842
        Tetrahydrobiopterin biosynthesis
      - M00843
        L-threo-Tetrahydrobiopterin biosynthesis
      - M00880
        Molybdenum cofactor biosynthesis
      - M00140
        C1-unit interconversion, prokaryotes
      - M00141
        C1-unit interconversion, eukaryotes
      - M00846
        Siroheme biosynthesis
      - M00868
        Heme biosynthesis, animals and fungi
      - M00121
        Heme biosynthesis, plants and bacteria
      - M00926
        Heme biosynthesis, bacteria
      - M00847
        Heme biosynthesis, archaea
      - M00924
        Cobalamin biosynthesis, anaerobic
      - M00925
        Cobalamin biosynthesis, aerobic
      - M00122
        Cobalamin biosynthesis
      - M00836
        Coenzyme F430 biosynthesis
      - M00117
        Ubiquinone biosynthesis, prokaryotes
      - M00128
        Ubiquinone biosynthesis, eukaryotes
      - M00116
        Menaquinone biosynthesis
      - M00930
        Menaquinone biosynthesis, futalosine pathway
      - M00931
        Menaquinone biosynthesis, modified futalosine pathway
      - M00932
        Phylloquinone biosynthesis
      - M00112
        Tocopherol/tocotorienol biosynthesis
      - M00933
        Plastoquinone biosynthesis
  - Biosynthesis of terpenoids and polyketides
    - Terpenoid backbone biosynthesis
      - M00095
        C5 isoprenoid biosynthesis, mevalonate pathway
      - M00849
        C5 isoprenoid biosynthesis, mevalonate pathway, archaea
      - M00096
        C5 isoprenoid biosynthesis, non-mevalonate pathway
      - M00367
        C10-C20 isoprenoid biosynthesis, non-plant eukaryotes
    - Plant terpenoid biosynthesis
      - M00097
        beta-Carotene biosynthesis
      - M00372
        Abscisic acid biosynthesis
      - M00371
        Castasterone biosynthesis
      - M00927
        Gibberellin A12 biosynthesis
      - M00928
        Gibberellin A4/A1 biosynthesis
      - M00929
        Gibberellin A1 biosynthesis
    - Macrolide biosynthesis
      - M00773
        Tylosin biosynthesis
      - M00934
        Mycinamicin biosynthesis
      - M00774
        Erythromycin biosynthesis
      - M00775
        Oleandomycin biosynthesis
      - M00776
        Pikromycin/methymycin biosynthesis
      - M00777
        Avermectin biosynthesis
    - Enediyne biosynthesis
      - M00824
        9-membered enediyne core biosynthesis
      - M00825
        10-membered enediyne core biosynthesis
      - M00826
        C-1027 benzoxazolinate moiety biosynthesis
      - M00827
        C-1027 beta-amino acid moiety biosynthesis
      - M00828
        Maduropeptin beta-hydroxy acid moiety biosynthesis
      - M00829
        3,6-Dimethylsalicylyl-CoA biosynthesis
      - M00830
        Neocarzinostatin naphthoate moiety biosynthesis
      - M00831
        Kedarcidin 2-hydroxynaphthoate moiety biosynthesis
      - M00832
        Kedarcidin 2-aza-3-chloro-beta-tyrosine moiety biosynthesis
      - M00834
        Calicheamicin orsellinate moiety biosynthesis
      - M00833
        Calicheamicin biosynthesis
    - Type II polyketide biosynthesis
      - M00778
        Type II polyketide backbone biosynthesis
      - M00779
        Dihydrokalafungin biosynthesis
      - M00780
        Tetracycline/oxytetracycline biosynthesis
      - M00823
        Chlortetracycline biosynthesis
      - M00781
        Nogalavinone/aklavinone biosynthesis
      - M00782
        Mithramycin biosynthesis
      - M00783
        Tetracenomycin C/8-demethyltetracenomycin C biosynthesis
      - M00784
        Elloramycin biosynthesis
    - Polyketide sugar unit biosynthesis
      - M00793
        dTDP-L-rhamnose biosynthesis
      - M00794
        dTDP-6-deoxy-D-allose biosynthesis
      - M00795
        dTDP-beta-L-noviose biosynthesis
      - M00796
        dTDP-D-mycaminose biosynthesis
      - M00797
        dTDP-D-desosamine biosynthesis
      - M00798
        dTDP-L-mycarose biosynthesis
      - M00799
        dTDP-L-oleandrose biosynthesis
      - M00800
        dTDP-L-megosamine biosynthesis
      - M00801
        dTDP-L-olivose biosynthesis
      - M00802
        dTDP-D-forosamine biosynthesis
      - M00803
        dTDP-D-angolosamine biosynthesis
  - Biosynthesis of other secondary metabolites
    - Biosynthesis of phytochemical compounds
      - M00039
        Monolignol biosynthesis
      - M00137
        Flavanone biosynthesis
      - M00940
        Flavanone biosynthesis
      - M00138
        Flavonoid biosynthesis
      - M00941
        Isoflavone biosynthesis
      - M00942
        Pterocarpan biosynthesis
      - M00966
        Equol biosynthesis
      - M00967
        Flavone degradation
      - M00962
        Psilocybin biosynthesis
      - M00963
        Chanoclavine aldehyde biosynthesis
      - M00964
        Fumigaclavine biosynthesis
      - M00965
        Vindoline biosynthesis
      - M00943
        Reticuline biosynthesis
      - M00944
        Morphine biosynthesis
      - M00945
        Sanguinarine biosynthesis
      - M00946
        Noscapine biosynthesis
      - M00961
        Betacyanin biosynthesis
      - M00370
        Glucosinolate biosynthesis
      - M00900
        Crocin biosynthesis
      - M00971
        QS-7 biosynthesis
      - M00894
        Cannabidiol biosynthesis
      - M00953
        Mugineic acid biosynthesis
      - M00952
        Benzoxazinoid biosynthesis
      - M00902
        Podophyllotoxin biosynthesis
    - Biosynthesis of beta-lactams
      - M00672
        Penicillin biosynthesis
      - M00673
        Cephamycin C biosynthesis
      - M00675
        Carbapenem-3-carboxylate biosynthesis
      - M00736
        Nocardicin A biosynthesis
      - M00674
        Clavaminate biosynthesis
    - Biosynthesis of other antibiotics
      - M00877
        Kanosamine biosynthesis
      - M00889
        Puromycin biosynthesis
      - M00815
        Validamycin A biosynthesis
      - M00904
        Dapdiamides biosynthesis
      - M00787
        Bacilysin biosynthesis
      - M00785
        Cycloserine biosynthesis
      - M00848
        Aurachin biosynthesis
      - M00788
        Terpentecin biosynthesis
      - M00819
        Pentalenolactone biosynthesis
      - M00903
        Fosfomycin biosynthesis
      - M00890
        Roseoflavin biosynthesis
      - M00951
        Cremeomycin biosynthesis
      - M00969
        Fumagillin biosynthesis
    - Biosynthesis of other fungal compounds
      - M00661
        Paspaline biosynthesis
      - M00786
        Fumitremorgin alkaloid biosynthesis
      - M00937
        Aflatoxin biosynthesis
      - M00893
        Lovastatin biosynthesis
      - M00891
        Ditryptophenaline biosynthesis
      - M00901
        Fumiquinazoline biosynthesis
    - Biosynthesis of other bacterial compounds
      - M00814
        Acarbose biosynthesis
      - M00789
        Rebeccamycin biosynthesis
      - M00790
        Pyrrolnitrin biosynthesis
      - M00805
        Staurosporine biosynthesis
      - M00808
        Violacein biosynthesis
      - M00835
        Pyocyanine biosynthesis
      - M00837
        Prodigiosin biosynthesis
      - M00838
        Undecylprodigiosin biosynthesis
      - M00921
        Cyclooctatin biosynthesis
      - M00905
        Grixazone biosynthesis
      - M00876
        Staphyloferrin A biosynthesis
      - M00875
        Staphyloferrin B biosynthesis
      - M00918
        Aerobactin biosynthesis
      - M00906
        Ethynylserine biosynthesis
  - Xenobiotics biodegradation
    - Aromatics degradation
      - M00538
        Toluene degradation
      - M00537
        Xylene degradation
      - M00419
        Cymene degradation
      - M00547
        Benzene/toluene degradation
      - M00548
        Benzene degradation
      - M00551
        Benzoate degradation
      - M00637
        Anthranilate degradation
      - M00568
        Catechol ortho-cleavage
      - M00569
        Catechol meta-cleavage
      - M00539
        Cumate degradation
      - M00543
        Biphenyl degradation
      - M00544
        Carbazole degradation
      - M00418
        Toluene degradation, anaerobic
      - M00541
        Benzoyl-CoA degradation
      - M00540
        Benzoate degradation
      - M00534
        Naphthalene degradation
      - M00638
        Salicylate degradation
      - M00624
        Terephthalate degradation
      - M00623
        Phthalate degradation
      - M00636
        Phthalate degradation
      - M00878
        Phenylacetate degradation
      - M00545
        Trans-cinnamate degradation
      - M00915
        Caffeine degradation


KGML

Image (png) file 1x
