# Supplementary material for: Metabolomic insights into variable antihistamine responses in allergic rhinitis: unveiling biomarkers for precision treatment
Source: Front Immunol. 2025 Jun 17;16:1565972. doi: 10.3389/fimmu.2025.1565972 (PMC12209198; doi:10.3389/fimmu.2025.1565972)
Supplement: Supplementary file 1 [file DataSheet1.zip › Supplementary file 2/ko00350.html]

KEGG PATHWAY: Tyrosine metabolism - Homo sapiens (human)


# Tyrosine metabolism - Homo sapiens (human)


[
Pathway menu
|
Organism menu
|
Pathway entry
|
Download
|
Help
]


##### Option

Scale:


100%

Image resolution:


High

##### Background color

Organism

Split cells that has orgs.
  


Exclude cells that has no orgs.

##### Search

##### ID search

##### Color

##### Module

Complete only

Including 1 block missing

Including any incomplete

- Pathway modules
  - Amino acid metabolism
    - Aromatic amino acid metabolism
      - M00042
        Catecholamine biosynthesis
      - M00043
        Thyroid hormone biosynthesis
      - M00044
        Tyrosine degradation
      - M00533
        Homoprotocatechuate degradation

##### Network

- nt06016
  Phenylalanine and tyrosine metabolism
  - N00708
    Tyrosine degradation
- nt06028
  Dopamine and serotonin metabolism
  - N01530
    Dopamine metabolism
  - N01550
    Adrenaline metabolism
  - N01552
    Eumelanin biosynthesis
- nt06322
  TRH-TSH-TH signaling
  - N00803
    Iodide organification/coupling reactions


KGML

Image (png) file 1x

Image (png) file 2x
