# Supplementary figures and images for: Metabolomic insights into variable antihistamine responses in allergic rhinitis: unveiling biomarkers for precision treatment
Source: Front Immunol. 2025 Jun 17;16:1565972. doi: 10.3389/fimmu.2025.1565972 (PMC12209198; doi:10.3389/fimmu.2025.1565972)

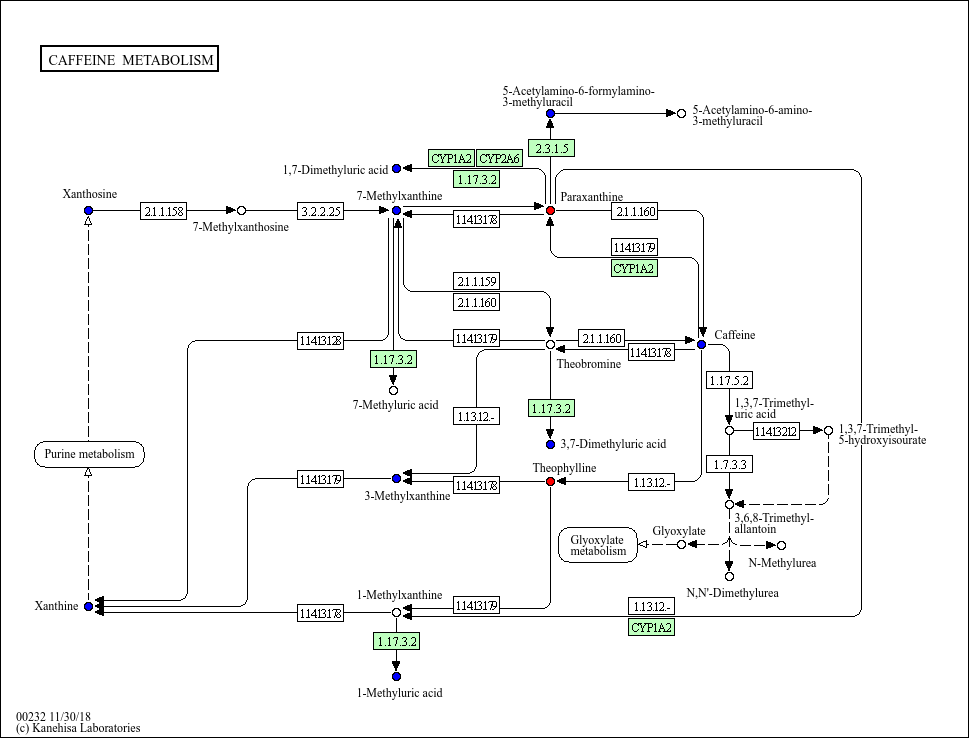

Supplement: Supplementary file 1 [file DataSheet1.zip › Supplementary file 2/ko00232.png]

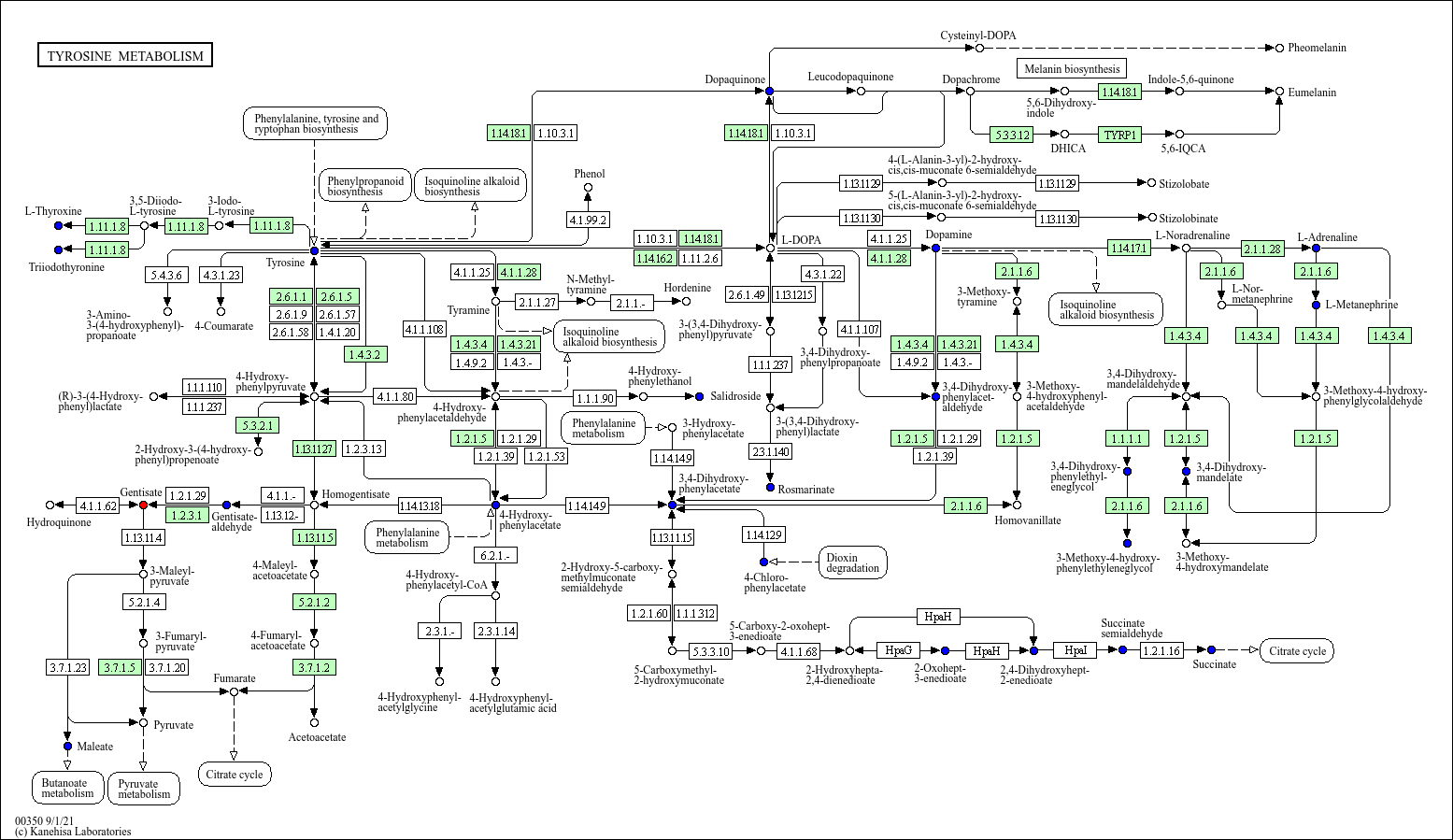

Supplement: Supplementary file 1 [file DataSheet1.zip › Supplementary file 2/ko00350.png]

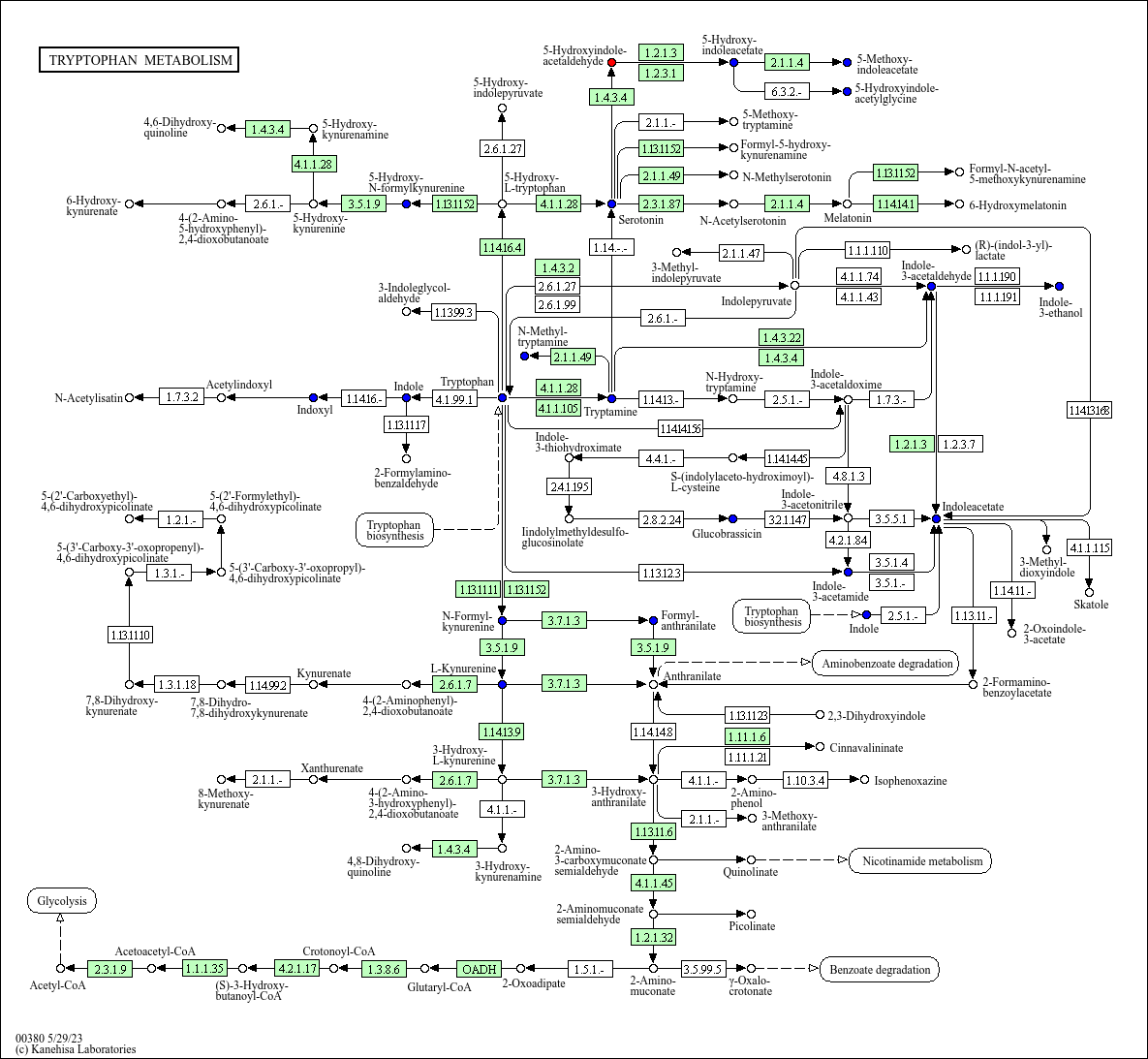

Supplement: Supplementary file 1 [file DataSheet1.zip › Supplementary file 2/ko00380.png]

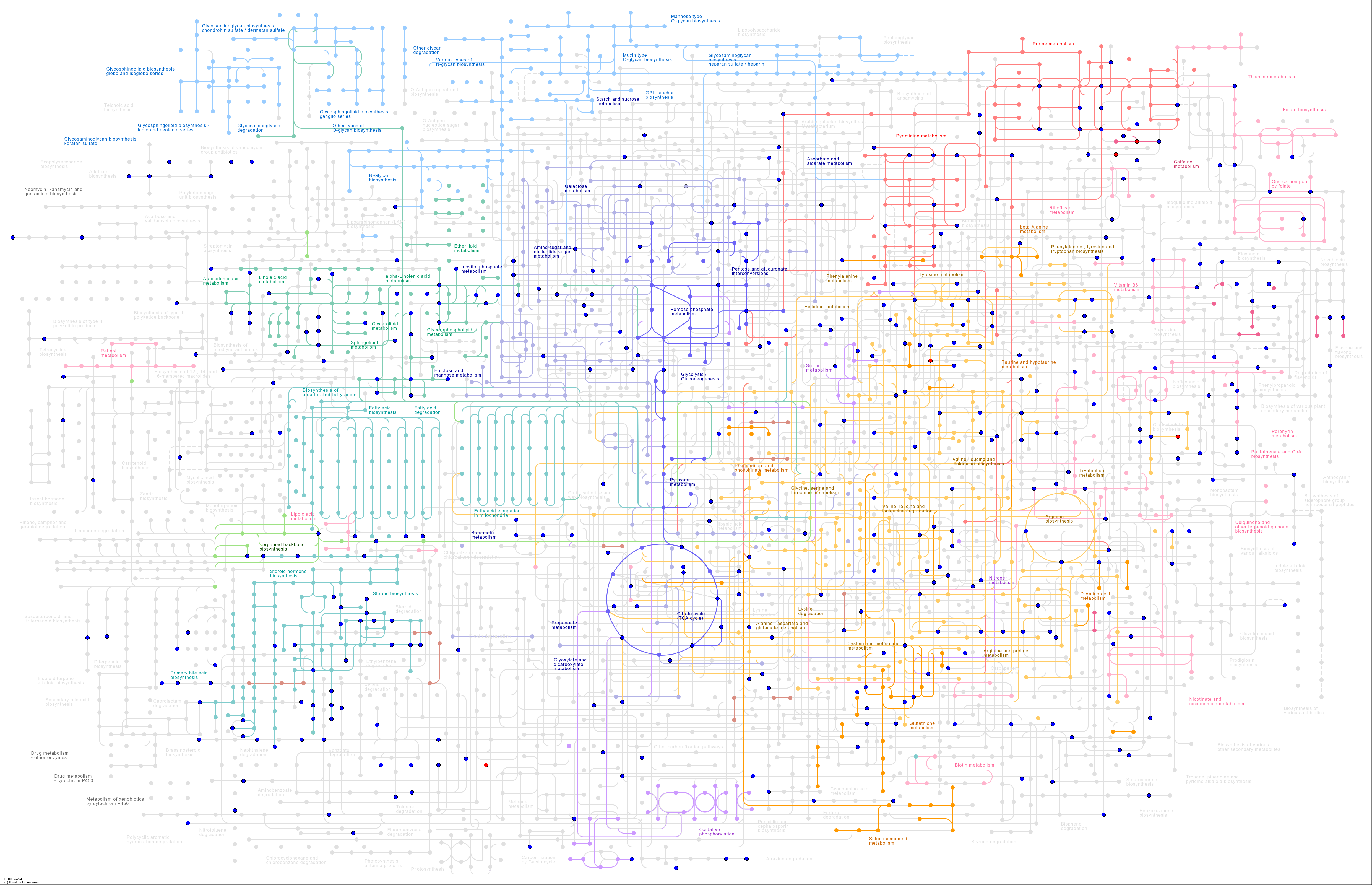

Supplement: Supplementary file 1 [file DataSheet1.zip › Supplementary file 2/ko01100.png]

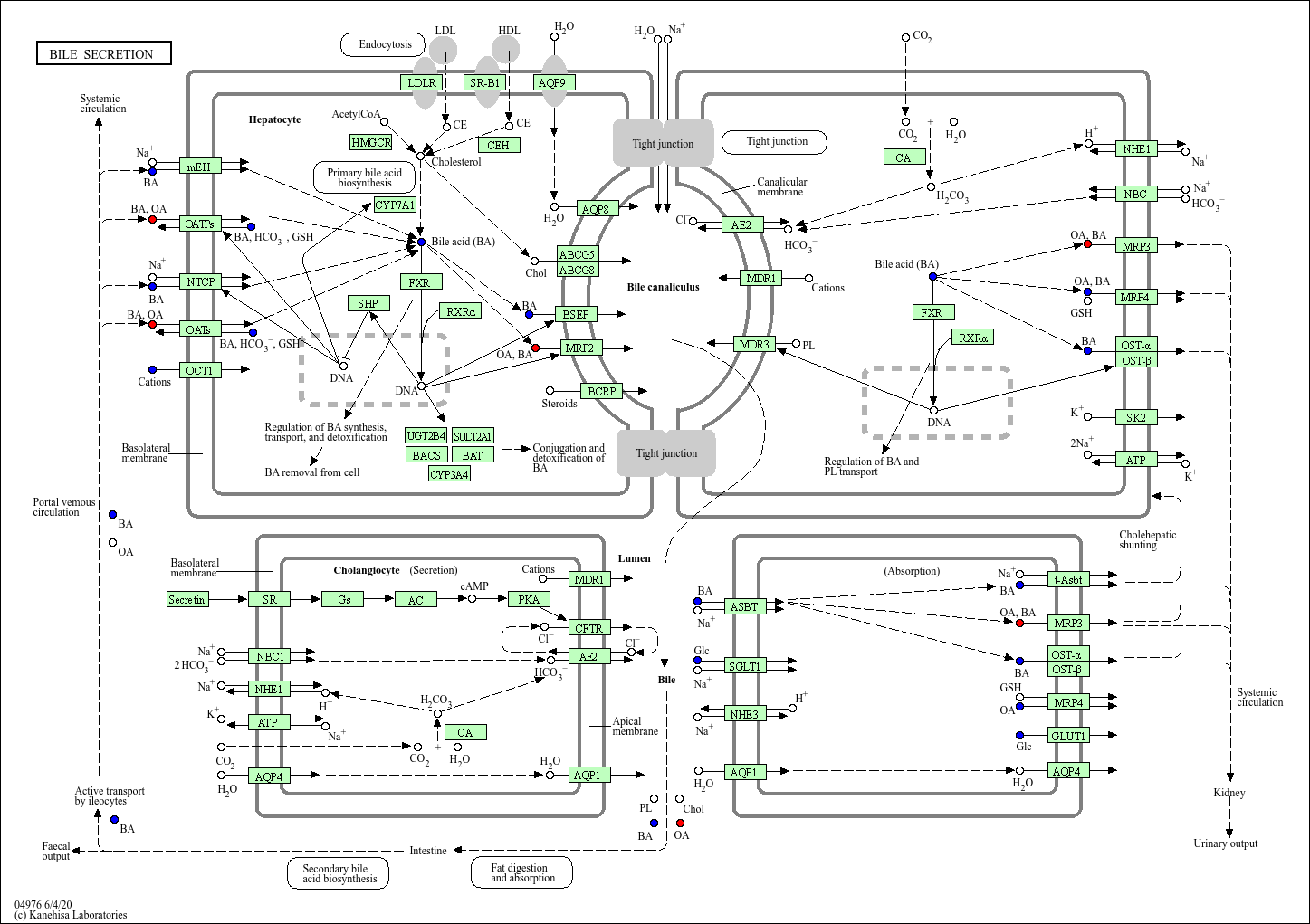

Supplement: Supplementary file 1 [file DataSheet1.zip › Supplementary file 2/ko04976.png]
